# The Impact of Malignancy on the Risk of Venous Thromboembolism in Pregnant Women: A Systematic Review

**DOI:** 10.1002/hsr2.70456

**Published:** 2025-04-10

**Authors:** Ali Vaezi, Seyyed Kiarash Sadat Rafiei, Bita Amiri, Ali Rezvanimehr, Maryam Naji abhary, Pariya Mahdavi, Mohammad Abbasalizadeh, Ghazale Yavari, Mahsa Shirforoush Sattari, Ali Kheirandish, Gisou Erabi, Foad Vakili Zadeh, Fatemeh Rasekh, Asiyeh Pormehr‐yabandeh, Seyede Zohreh Mohagheghi, Hoda Zaraj, Amir Abdi, Parisa alsadat Dadkhah, Niloofar Deravi

**Affiliations:** ^1^ Student Research Committee, School of Medicine Tehran University of Medical Sciences Tehran Iran; ^2^ Student Research Committee, School of Medicine Shahid Beheshti University of Medical Sciences Tehran Iran; ^3^ Cardiovascular Research Center Tabriz University of Medical Sciences Tabriz Iran; ^4^ Network of Immunity in Infection, Malignancy and Autoimmunity (NIIMA) Universal Scientific Education and Research Network (USERN) Tehran Iran; ^5^ Faculty of Medicine, Tehran Medical Sciences Branch Islamic Azad University Tehran Iran; ^6^ Department of Midwifery, School of Nursing & Midwifery Mashhad University of Medical Sciences Mashhad Iran; ^7^ Student Research Committee Tabriz University of Medical Sciences Tabriz Iran; ^8^ Research Center for Evidence-Based Medicine, Iranian EBM Centre: A JBI Centre of Excellence, Faculty of Medicine Tabriz University of Medical Sciences Tabriz Iran; ^9^ Student Research Committee, School of Medicine Shahroud University of Medical Sciences Shahroud Iran; ^10^ School of Medical Sciences Azad Sari University of Medical Sciences Mazandaran Iran; ^11^ Student Research Committee, Faculty of Pharmacy Mazandaran University of Medical Sciences Sari Iran; ^12^ Student Research Committee Urmia University of Medical Sciences Urmia Iran; ^13^ Student Research Committee, School of Medicine Guilan University of Medical Sciences Rasht Iran; ^14^ Student Research Committee, School of Medicine Shiraz University of Medical Science Shiraz Iran; ^15^ Hormozgan Health Institute Hormozgan University of Medical Science Bandar Abbas Iran; ^16^ School of Medicine Iran University of Medical Sciences Tehran Iran; ^17^ Tehran University‐Caspian Campus Tehran Iran; ^18^ Student Research Committee, School of Medicine, Tehran Medical Sciences Islamic Azad University Tehran Iran; ^19^ School of Medicine Isfahan University of Medical Sciences Isfahan Iran

**Keywords:** cancer, pregnancy, pregnant women, risk of venous thromboembolism, venous thromboembolism

## Abstract

**Background and Aims:**

Venous thromboembolism (VTE) is a distinct malignancy complication that raises the risk of demise in cancer patients by up to thrice. However, pregnant females have a 4–5 times greater chance of getting VTE than nonpregnant women. The current systematic review aimed to elucidate the impact of malignancy on the risk of VTE in pregnant females.

**Methods:**

We carried out a systematic search in multiple databases, including PubMed (Medline), Google Scholar, and Scopus, up to January 2023. Finally, 441 related articles were extracted from the databases. After screening the title, abstract, and full text, seven articles were included in the study.

**Results:**

Seven studies (six cohorts and one cross‐sectional) with 58,854,195 pregnant females (22,396 cancer patients) were included. These studies were done in the United States of America, Canada, Brazil, and Denmark. All of the studies except one study demonstrated that cancer in pregnant patients increased the risk of deep vein thrombosis (DVT). The risk of VTE prevalence in pregnant females with a record of malignancy was significantly higher than in free cancer groups, and the highest aOR was correlated to myeloid leukemia.

**Conclusions:**

Evidence in this systematic review showed that pregnant women with malignancy are more susceptible to VTE and other coagulation disorders. Physicians and health policymakers should be of high vigilance to pregnancy‐associated VTE, especially in women who have cancer.

## 1 Introduction

Malignancy is a disease in which some cells present uncontrolled divisions and dissipate to destroy other tissues throughout the body. Venous thromboembolism (VTE) contains Venous thrombosis (if a thrombus develops in a large vein) and pulmonary embolism (the transfer of a loosed clot through the bloodstream to the lungs) [1]. It has been assessed that 20% of these demises happen in patients with malignancy [2]. According to recent research, patients with cancer and VTE have a mortality rate much more than three times greater than many without this VTE [3].

The correlation between cancer, pregnancy, and VTE is rooted in distinct biological processes that increase the risk of blood clot formation. Pregnancy induces substantial physiological changes, leading to elevated levels of coagulation‐promoting factors, thereby predisposing individuals to VTE [4]. Elyamany et al suggested that cancer induces a hypercoagulable state by releasing pro‐inflammatory cytokines and tissue factors from tumor cells, and this leads to an increased incidence of thrombotic events [5]. Hamza et al. study showed that, in individuals with concurrent pregnancy and cancer, the risk of VTE is notably elevated due to the concurrent stimulation of increased blood coagulation and vascular damage by both conditions [6].

The pathogenetic mechanisms of thrombosis include a complex interaction between three pillars: 1‐ tumor cells, 2‐ the hemostatic system, 3‐ properties of each individual [7]. Evidence indicates that the overall risk varies depending on the kind of cancer, the degree or severity of the malignancy, and, of course, the therapy with antineoplastic medicines. Malignancy and its therapies are known risk factors for VTE [8]. In comparison to nonpregnant females, pregnant women have a 4–5 times developed risk of rising VTE [9]. Several studies have found that enceinte ladies with malignancy are far more likely than those without cancer to experience pregnancy‐related VTE [9]. So, the interaction between pregnancy and cancer impact of thromboembolism is too important for saving the mother and her newborns. This is the first review study to consider three pregnancy, cancer, and VTE factors.

## 2 Material and Methods

The current systematic review investigates the connection between pregnant women's chance of VTE and cancer deaths. The design procedure was recorded in OSF (Open Science Framework, Registration https://doi.org/10.17605/OSF.IO/3ZGJF), and the ethical statement does not apply to this systematic review. This study's search strategy, screening, and data selection were checklist‐based. PRISMA (Preferred Journalism Substances for Systematic Reviews and Meta‐analyses) was observed.

### 2.1 Search Plan

A comprehensive search was shown up to January 2023 in PubMed (Medline), Scopus, and Google Scholar databases based on the title/abstract, using the advanced search strategy, suitable operators, and tags for each database. The search strategy of the Mesh database is summarized in Table 1. Thus, as the first step, the studies were searched for and retrieved. In the next step, after removing duplicates, two researchers evaluated the titles and full texts of the obtained studies. Studies that matched the eligibility requirements were found and added to the analysis.

**Table 1:** Search strategy of PubMed, Google Scholar, and Scopus databases.

Searched keywords	Up to	Database/result number
((pregnancy[Title/Abstract]) OR (pregnant[Title/Abstract])) AND ((cancer[Title/Abstract]) OR (neoplasm[Title/Abstract]) OR (glioma[Title/Abstract]) OR (glioblastoma[Title/Abstract]) OR (“Acute leukemia”[Title/Abstract])OR (Leukemia [Title/Abstract]) OR (Melanoma [Title/Abstract])OR (“Malignant melanoma”[Title/Abstract]) OR (“breast cancer”[Title/Abstract]) OR (“Cervical cancer”[Title/Abstract]) OR (“Ovarian cancer”[Title/Abstract]) OR (“Gastrointestinal cancer”[Title/Abstract]) OR (“Thyroid cancer”[Title/Abstract]) OR (“CNS cancer”[Title/Abstract]) OR (“Central nervous system cancer”[Title/Abstract]) OR (“Hodgkin's lymphoma “[Title/Abstract]) OR (“Lymphoid leukemia”[Title/Abstract]) OR (“Myeloid leukemia”[Title/Abstract]) OR (“tissue sarcoma”[Title/Abstract]) OR (“Carcinoid tumor”[Title/Abstract]) OR (“Adrenal cancer”[Title/Abstract]) OR (“Peritoneal cancer”[Title/Abstract]) OR (“Colon cancer”[Title/Abstract]) OR (“lung cancer”[Title/Abstract]) OR (“Uterus cancer”[Title/Abstract]) OR (“liver cancer”[Title/Abstract]) OR (“stomach cancer”[Title/Abstract])) AND ((DVT[Title/Abstract]) OR (venous thromboembolism[MeSH Terms]) OR (pulmonary embolism[MeSH Terms]))	January/2023	**PubMed:** 157
allintitle: (Pregnancy OR pregnant) AND (Cancer OR neoplasm) AND (“venous thromboembolism” OR “pulmonary embolism” OR “VTE”)	January/2023	**Google Scholar:** 7
TITLE‐ABS (pregnancy OR pregnant) AND (Cancer OR neoplasm OR glioma OR glioblastoma OR Leukemia OR Melanoma OR “Acute leukemia” OR “Malignant melanoma” OR “breast cancer” OR “Cervical cancer” OR “Ovarian cancer” OR “Gastrointestinal cancer” OR “Thyroid cancer” OR “CNS cancer” OR “Central nervous system cancer” OR “Hodgkin's lymphoma” OR “ Lymphoid leukemia” OR “Myeloid leukemia” OR “tissue sarcoma” OR “Carcinoid tumor” OR “Adrenal cancer” OR “Peritoneal cancer” OR “Colon cancer” OR “lung cancer” OR “stomach cancer”) AND (“venous thromboembolism” OR “pulmonary embolism” OR DVT)	January/2023	**Scopus:** 277

### 2.2 Inclusion and Exclusion Criteria

The scholarly works that comprised the current research examined the risk of VTE in pregnant females with malignancy, which comprised observational studies. The results of VTE in pregnant people with a history of malignancy were also examined. Interventional studies, reviews, case studies, letters to the editor, posters, and abstracts were all excluded.

We predefined the inclusion criteria for eligible studies as follows: 1‐ original studies published up to January 2023, 2‐ English language articles published in peer‐reviewed journals, 3‐ observational studies with case‐control, cross‐sectional, and longitudinal cohort designs, and 4‐ articles reported incidence data and incidence rates separately, or when they provided sufficient data to allow calculations for the postoperative VTE incidence among pregnant patients.

Our exclusion criteria follow these standards: 1‐ articles published as conference abstracts, commentary, case reports, narrative reviews, series case reports, editorials, letters, and short communications, 2‐ studies with small sample size, 3‐ studies with who only reported the incidence of VTE and 4‐ when multiple studies reported data from the same study population were encountered, only the most comprehensive and accurate studies were included, and others were excluded.

### 2.3 Quality Assessment

The Newcastle‐Ottawa checklist was recycled in Table 3 to measure the quality of studies. The full text of the studies was assessed for quality to investigate them all, and the ones that met the inclusion criteria were evaluated by two people independently. If there was a difference of opinion, a consensus was reached. After that, two separate study team members produced the data extraction form and extracted the data. The information extracted from the extraction form comprised the study type, location, sample size, type of cancer, and the result of VTE incidence.

## 3 Results

### 3.1 Study Selection

**Figure 1:**
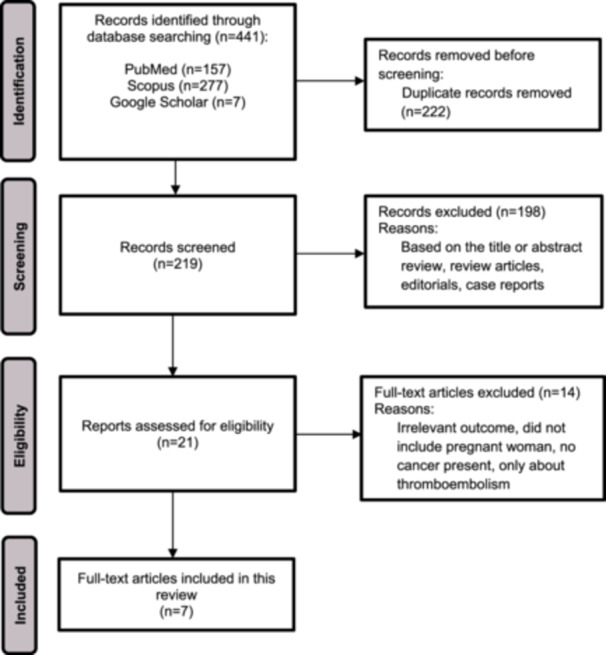
PRISMA flow diagram of the study selection in this systematic review.

We performed a thorough search in three databases up to January 2023. Overall, 441 articles were selected through PubMed (Medline), Scopus, and Google Scholar, of which 222 copies were detached. Of the residual 219 records, 198 were excluded for review articles, case reports, and based on the title or abstract review. Finally, 14 pieces were removed after full‐text assessment, and seven eligible studies were involved in this review. See the PRISMA flow diagram for more information on the exclusion criteria (see Figure 1).

### 3.2 Study Characteristics

This review is comprised of six studies [10, 11, 12, 13, 14, 15] investigating the risk of VTE incidents in pregnant females with and without antiquity of cancer and one study [16] that assessed pregnant women with cancer for VTE risk based on the type of given treatments. Three of the seven studies that made up this evaluation were carried out in the United States [10, 14, 15], two in Canada [12, 13], one in Brazil [16], and one in Denmark [11]. Regarding the study design, there were four retrospective cohorts [10, 12, 13, 15], one prospective [16], and one historical cohort [11] studies alongside one cross‐sectional study [14]. All of the included reports were published between 2016 and 2022.

As to the participants, a total of 58,854,195 pregnant women had been enrolled in these studies, of which 22,396 patients had a history of a specific type of cancer. It is important to note that three studies [12, 13, 15] used the same population of pregnant women (*n* = 14,513,587) as their study sample. These studies acquired their study sample from the Health‐Care Cost and Utilization Project‐Nationwide Inpatient Sample (HCUP‐NIS) between 1999 and 2014. Three studies [11, 14, 16] Provided the mean age of their participants, which ranged from 27.3 to 32.9, while in three other studies [12, 13, 15] No details were provided regarding their patients' mean age, but their participants were mainly between 25 and 34 years old. Unfortunately, one study [10] did not detail the age of its patients, and only one study gave information on the gestational age of the participants. The follow‐up duration (excluding one cross‐sectional study [14]) ranged from 3 months to 40 years (median [Q1–Q3] 15 [6, 7, 8, 9, 10, 11, 12, 13, 14, 15, 16, 17, 18, 19, 20, 21, 22]) in the included records [10, 11, 12, 13, 15, 16]. All of the studies considered VTE as their type of embolism (Table 2).

**Table 2:** Summary of the characteristics of the included studies.

Study	Year	Country	Study design	Follow‐up (years)	Time of data collection	Participants		Mean age (SD)	Type of cancer (%)	Type of embolism	VTE Prevalence (%)		VTE risk (aOR, [95% CI])	*p* value	Quality score
						Total (*n*)	Cancer (*n*)				Without cancer	With cancer			
Nolan et al. [12]	2019	Canada	Retrospective cohort	15	1999–2014	14,513,587	291	NR	Acute leukemia	VTE	0.34	5.8	10.09 [5.87–17.33]	*p* < 0.0001	6/11
Greiber et al. [11]	2020	Denmark	Historical cohort	40	January 1977–December 2017	3,581,214	4268 (Cancer in pregnancy group = 1330, prepregnancy group = 2938)	30.83 (5.13)	Melanoma (27.2), breast (17.2), Cervical (14.7), Ovarian (4), Gastrointestinal (3.9), Thyroid (3.4), Central nervous system (3.1), Hodgkin's lymphoma (2.4), Leukemia (2.9), Non ‐Hodgkin's lymphoma (1.1), Other (20.2)	VTE	0.1074	(Cancer in pregnancy group: 0.7519, prepregnancy group: 0.2383)	Patients with cancer diagnosis during pregnancy (*n* = 1330): 6.50 [3.48–12.12] Patients with cancer diagnosis within 2 years before pregnancy (*n* = 2938): 1.99 [0.95–4.19]	*p* = 0.023 *p* = 0.075	9/11
Bleau et al. [10]	2016	USA	Retrospective cohort	8	2003–2011	7,917,453	2826	NR	Hodgkin's disease (22.5), Breast (20), Non ‐Hodgkin's lymphoma (15.4), (9), Thyroid (7), Lymphoid leukemia (6.2), Myeloid leukemia (6), Malignant melanoma (4.4), Ovarian (4.2), Brain (3.9)	VTE	NR	NR	Myeloid leukemia = 20.75 [6.61–65.12] Ovarian cancer = 10.35 [1.44–74.19] Cervical cancer = 8.64 [2.15–34.79] Hodgkin's disease = 7.87 [2.94–21.05]	*p* < 0.0001 *p* = 0.04 *p* = 0.007 *p* < 0.0001	9/11
Nolan et al. [13]	2019	Canada	Retrospective cohort	16	1999–2014	14,513,587	1269	NR	Leukemia	VTE	0.34	1.65	4.40 [2.86–6.78]	*p* < 0.0001	9/11
Hase et al. [16]	2018	Brazil	Prospective cohort	3 months	December 2014–July 2016	NA	52	31.04 (6.33) (high risk score = 32.9(6.27), low risk score = 27.3 (4.75))	Breast (44.2), Cervical (17.3), Leukemia(9.6), Lymphoma(7.7), Gastrointestinal (3.8), Bone (3.8), Ovarian (3.8), tissue sarcoma(3.8), Carcinoid tumor (1.9), Soft Thyroid (1.9), Adrenal (1.9)	VTE	0.00	0.00	No patient exhibited VTE in either groups. (thomboprophylaxis receiving group or ambulation group)	NA	7/11
Spiegel et al. [15]	2018	USA	Retrospective cohort	15	1999–2014	14,513,587	581	NR	Thyroid cancer	VTE	0.32	0.86	2.42 [1.00–5.84]	*p* = 0.04	8/11
Rubens et al. [14]	2022	USA	Cross‐sectional	NA	2010–2014	3,814,715	13,109	Cancer survivors: 30.5 (0.08) Without cancer: 28.0 (0.03)	Lymphomas and leukemia (31.3), Other female genital organs (27.2), Urinary (15.5), Breast (9.2), Ovary (2.4), Melanomas of skin (1.8), GI organs and peritoneum (0.99), Colon (0.93), Lung and bronchus (0.5), Uterus (0.42), Liver (0.24), Stomach (0.18), Cancer with unspecified primary (8.4), Secondary malignancies (0.94), Malignant neoplasm without specification of site (0.09)	VTE	0.21	1	3.62 [2.69–4.88]	*p* < 0.0001	7/8

Abbreviations: aOR, adjusted odd ratio; CI, confidence interval; NA, not appropriate; NR, not recorded; SD, standard deviation; VTE, venous thromboembolism

**Table 3:** Newcastle ‐ Ottawa quality assessment scale.

Study	Selection				Comparability	Outcomes			Total
	Representativeness of exposed cohort	Selection of nonexposed cohort	Ascertainment of exposure	Outcome not present at the start of the study		Assessment of outcomes	Length of follow‐up	Adequacy of follow‐up	
Nolan et al. [12]	*	*	*	*	*	*	*	*	********
Greiber et al. [11]	*	*	*	*	*	*	*	*	********
Bleau et al. [10]	*			*	*	*	*	*	******
Nolan et al. [13]	*	*	*	*	*	*	*	*	********
Hase et al. [16]	*	*	*	*	**	*	*	*	*********
Spiegel et al. [15]	*	*	*	*	**	*	*	*	*********
Rubens et al. [14]	*	*	*	*	**	*			*******

### 3.3 VTE Prevalence in Pregnant Females With and Without a Record of Cancer

In five studies [11, 12, 13, 14, 15] the VTE prevalence ranged from 0.2383% to 5.8% (median [Q1–Q3) 0.86 [0.23–1.65]) among the groups with a history of cancer and 0.1074% to 0.34% (median [Q1–Q3] 0.26 [0.08–0.34]) among the cancer‐free groups. One study did not provide any prevalence rates for VTE [10] and another study [16] had no VTE events at all. Overall, the incidences of VTE events were higher in the groups with a history of cancer compared with the cancer‐free groups. For more information, see Table 2.

### 3.4 VTE Risk in Pregnant Females With and Without a Record of Cancer

As a whole, in the comparison of pregnant females with a record of malignancy with cancer‐free ones, the median (Q1–Q3) of adjusted odds ratio (aOR) for VTE in six of the reviewed studies with significant results was 7.87 (4.01–10.22) (range 2.42–20.75) [10, 11, 12, 13, 14, 15]. Moreover, one of the studies [16] took a different approach, which is discussed at the end of this section.

Almost half of the reports [12, 13, 15] had only one specific type of cancer among their cancer group, while the other studies [10, 11, 14, 16] included patients with a variety of cancers. Also, only one study reported cancer‐specific VTE risk aORs [10].

As mentioned, three studies [12, 13, 15] evaluated patients with only one type of cancer. These studies used the same patients and database (HCUP‐NIS) for their study as well. Two of them also had the same authors, and they conducted their study on leukemic pregnant women, which resulted in an aOR of 10.09 (95% CI: 5.87–17.33) [12] and 4.40 (95% CI: 2.86–6.78) [13]. The other study [15], also using the mentioned population, registered pregnant women with thyroid cancer and obtained an aOR of 2.42 (95% CI: 1.00–5.84) for VTE event risk.

Regarding the four studies [10, 11, 14, 16] that covered various types of cancers, two studies [11, 14] simply reported an overall aOR for their study and not a cancer‐specific aOR. One of which [11] divided their patients (*n* = 4268) into two groups (one was comprised of women whose cancer was diagnosed 2 years before pregnancy [prepregnancy cancer, *n* = 2938] and the other whose cancer was diagnosed during pregnancy [cancer in pregnancy, *n* = 1330]) and reported group‐specific aORs (See Table 2 for the corresponding aORs). One of the two studies [10] with a cancer‐specific aOR reported cancer‐specific VTE risk aORs (adjusted odds ratio mean 11.9 ± 5.9). Interestingly, in this study, certain types of cancers, namely, head cancer, thyroid malignancy, melanoma, and lymphoid leukemia, developed no VTE events and, therefore, did not elevate the risk of VTE occurrence. Also, in this study, the *p* values for breast cancer and non‐Hodgkin's lymphoma were not significant. Moreover, the highest aOR among all studies was recorded in this study and was correlated to myelogenous leukemia with 20.75 (95% CI: 6.61–65.12) [10]. Lastly, the other of the two studies [16] conducted a survey, unlike the others. This study collected data from a clinical trial [17] in which it divided pregnant women with a variety of cancers (*n* = 52) into two high or low‐risk groups based on VTE risk scores that they calculated using the strategies of the Royal College of Obstetricians and Gynaecologists. Furthermore, they treated the high‐risk group with thromboprophylaxis and only allowed ambulation for the low‐risk group. This study followed up and compared their patients' outcomes for 3 months, resulting in no VTE events.

Altogether, most of the reviewed studies exhibited that a record of malignancy can moderately raise the risk of VTE events in pregnant women meaningfully.

## 4 Discussion

In this systematic review, we investigated seven articles, including six cohorts and one cross‐sectional study, around the influence of cancer on the risk of VTE in pregnant women. The articles had different results based on the design, duration, type of population (sex‐age‐race), and herbal preparation. Most articles showed that cancer in pregnant women increased the risk of VTE [10, 11, 13, 14, 15, 18] but one of the studies [16] showed no significant impact of cancer in increasing the VTE risk in pregnant women.

Earlier revisions have shown that coagulation factors collected in immobilized blood can override local anticoagulation mechanisms and induce a hypercoagulable state of cancer that may cause thromboembolism [19]. Normal pregnancy is carried out by increasing the absorption of von Willebrand factor and factors of X, VIII, VII, and significantly growing fibrinogen [20] to stop extreme hemorrhage throughout the end or after delivery. Therefore, the risk of VTE rises with pregnancy and malignancy.

Hypoxia and other triggers stimulate endothelial cells, which produce adhesion receptors such as von Willebrand factor, endothelial selectin, and platelet selectin that attach to leukocytes (mainly neutrophils and monocytes), microparticles (MPs), and platelets [21, 22, 23]. In normal conditions, the endothelium stops coagulation by preventing the binding of proteins and cells essential for coagulation, the expression of anticoagulants (thrombomodulin including tissue factor pathway inhibitor, heparin‐like proteoglycans, and endothelial protein C receptors), and the release of platelet inhibitors (nitric oxide and prostacyclin) [24].

Spiegel et al. [15] discovered that pregnant individuals with thyroid malignancy have a more significant occurrence of VTE than pregnant patients without thyroid malignancy. Patients with thyroid malignancy did not have a greater risk of postoperative complications than those deprived of thyroid malignancy, indicating that a more significant percentage of blood donations in these patients was attributable to the delivery technique.

A meta‐analysis conducted by Gajic‐Veljanoski et al. [25] in adult nonpregnant populations with VTE, cancer, and other underlying comorbidities revealed that long‐term low‐molecular‐weight heparin (LMWH) therapy resulted in a reduction in mean bone mineral density (BMD) from 1.2% to 2.8% to 4.8% after 3–24 months of use in two prospective observational studies of VTE prophylaxis with LMWH. Five clinical trials, however, did not show significant changes. The meta‐analysis did not indicate an increased risk of fractures with LMWH compared to control groups, mainly using unfractionated heparin (UFH) or vitamin K antagonists [25].

VTE was more shared in pregnant females with acute leukemia (ALS), according to Nolan et al. [18]. PA‐AL (pregnancy‐associated ALS) increased the risk of intrauterine fetal death in pregnant females. Gynecological and Breast malignancies increase the risk of VTE. Chemotherapy, metastasis, and central venous catheters aggravate the situation for these women [26].

We also consider that chemotherapy and radiation can damage sexual organs, including the uterus and ovaries, causing ovum mutations that can cause congenital disabilities and coagulation issues in the mother and fetus [27]. Khorana et al. [28] found a substantial link between chemotherapy‐induced VTE and higher platelet counts. Paracrine interplays between platelets and malignant cells facilitate malignant cells' distribution, survival, and expansion into circulation. The α‐granules of platelets have a provenance of transforming growth factor β, a secreted protein that controls cell growth, differentiation, proliferation, and apoptosis. Platelet‐derived growth factor is a protein in cell division and growth and promotes angiogenesis. The two prior proteins withhold host immunosurveillance and have hence been implicated in promoting tumor metastasis [29, 30]. Hase et al. [16] found that chemotherapy within 6 months was the most significant powerful risk factor for anticoagulation and VTE. Esposito et al. [31], showed that only the administration of chemotherapy during the fetal stage of the conceptus is very hazardous and can result in the termination of the pregnancy. When the disorder is diagnosed in the second or third trimester of gestation or when it is likely to delay the starting of chemotherapy beyond the 14th week, the risk of severe problems for the fetus is low, and pregnancy termination is not required. As a result, good cancer therapy with the patient situation is critical in reducing VTE mortality and morbidity and future studies should focus on that.

Bleau et al. [10] found that pregnant women with Hodgkin's disease and myeloid leukemia had a higher VTE risk. Hematological malignancies necessitate thromboprophylaxis because pregnancy and malignancy enhance coagulation factors. Hase et al. [16] stated that with thromboprophylaxis in inpatient pregnant women with cancer, VTE prevalence was lowered and did not arise during hospitalization or the 3 months following hospital birth. Thromboprophylaxis in nonhospitalized pregnant women, the collective mechanical route of pregnancy and malignancy in VTE, and longer‐term thromboprophylaxis should be studied.

As a result of hormonally induced declined venous capacitance and declined venous outflow, pregnant women are more likely at risk of VTE [32] and women who had high circulating estrogen levels during pregnancy or were subjected to the synthetic estrogen diethylstilbestrol are at greater risk of developing breast cancer [33] hence, we can conclude that hormonal change during pregnancy is an important risk factor for increasing both VTE and cancer in pregnant women. The other important risk factors include: 1‐thrombophilia 2‐ history of thrombosis 3‐ certain medical conditions 4‐ some complications of pregnancy and parturition [34].

According to Greiber et al. [11], VTE events were most frequent at the time of delivery (5.62 events per 10,000 pregnancies) and 1 month postpartum (1.72 events per 10,000 pregnancies). The third trimester showed more VTE incidences than the other two trimesters (ranging from 1.08 to 1.21 events per 10,000 pregnancies). This applies to pregnant women with a history of cancer. Similarly, previous studies on pregnant women without a cancer history found that VTE risk, despite being high during the pregnancy and the postpartum period, is significantly higher during the first 6 weeks of the postpartum period [35].

To the best of our knowledge, data regarding the manner of pregnancy (such as the use of ovulation‐stimulation drugs, etc.) has been neglected in previous studies, especially since they can increase VTE risk and act as a confounding factor. We recommend that future trials focus on this aspect.

Unfortunately, the evidence presented in this review does not encompass pertinent studies on the risk of VTE in pregnancies among individuals with cancer in Asia, and since this region has been established as high risk (risk factors such as sedentary lifestyle, obesity, etc.) for VTE risk [36], we suggest further studies be performed in this region as strong environmental factors are present.

### 4.1 VTE Prophylaxis in Pregnant Women With Cancer

Women at risk for VTE in pregnancy should be treated with heparin thromboprophylaxis beginning as early in pregnancy as is applied. Heparin is the anticoagulant drug option for VTE prophylaxis during pregnancy [37, 38].

Normally, many cases of VTE are managed according to well‐defined protocols using UFH or low molecular weight heparin (LMWH). Data gained from a huge number of nonpregnant patients, such as the overall clinical experience in pregnant women over the prior decade, support the use of LMWH in pregnant patients, and it should be the first line of treatment in pregnant women [39].

Compared to LMWH, UFH has the drawback of requiring hospitalization for at least several days to supervise the activated partial thromboplastin time (aPTT) and adjust the dose of UFH [40]. Nevertheless, UFH has one major benefit over other anticoagulants: it is fully reversible with protamine sulfate, making UFH a wise anticoagulant choice in some clinical settings during pregnancy [41]. There are two recognized regimens for management using a continuous intravenous infusion of unfractionated heparin. For these two regimens, it is so necessary to get an aPTT before starting heparin [37].

A small part of patients treated with UFH showed heparin resistance, perhaps due to increased heparin clearance, increased levels of factor VIII, or increased heparin‐binding proteins [42]. Although infrequent, antithrombin deficiency also results in an apparent lack of the usual heparin effect on the aPTT.

Pregnancy presumably increases renal clearance of LMWH, and other as yet poorly determined pregnancy‐related alterations in LMWH metabolism may be at play [43]. Depending upon the risk of VTE, supervision of LMWH function should be performed fairly frequently in pregnancy, perhaps at least once every 2‐4 weeks in most patients [44]. Many experts suggested supervising peak LMWH anti‐Xa levels in pregnant women to achieve levels of 0.6–1.2 U/mL at 3–3.5 h after SC injection [45]. Until now, the exact duration of acute VTE treatment with LMWH in pregnant women doesn't exist and future studies should focus on this.

Enoxaparin is likely the most widely used LMWH product in pregnancy in the United States. it is dosed at 1 mg/kg SC BID. Twice‐daily dosing of enoxaparin is superior to once‐daily dosing in terms of sustaining the targeted circulating levels of LMWH activity [43, 46].

Despite the increased risk of thrombosis during pregnancy and the postpartum period, most women do not necessitate anticoagulation Except pregnant women with: 1‐ current thrombosis, 2‐ a history of thrombosis, 3‐ thrombophilia, 4‐ history poor pregnancy outcome, and 5‐ women at high risk for thrombosis postpartum [34].

**Figure 2:**
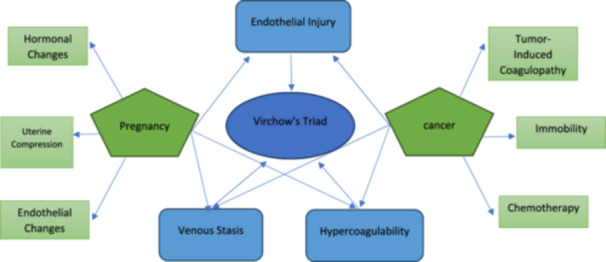
This diagram illustrates the pathophysiology of VTE in the context of pregnancy and cancer: Virchow's Triad consists of three main components: endothelial injury, venous stasis, and hypercoagulability, all of which contribute to the development of thrombosis. These elements are present in both pregnancy and cancer, where they interact with specific factors. In pregnancy, endothelial injury, venous stasis, and hypercoagulability are influenced by hormonal changes, uterine compression, and endothelial alterations. In cancer, these triad components are further exacerbated by tumor‐induced coagulopathy, immobility, and chemotherapy treatments. Both pregnancy and cancer increase the risk of thrombosis by affecting the components of Virchow's Triad, creating a complex interplay between the body's physiological changes and the development of blood clots [47].

**Figure 3:**
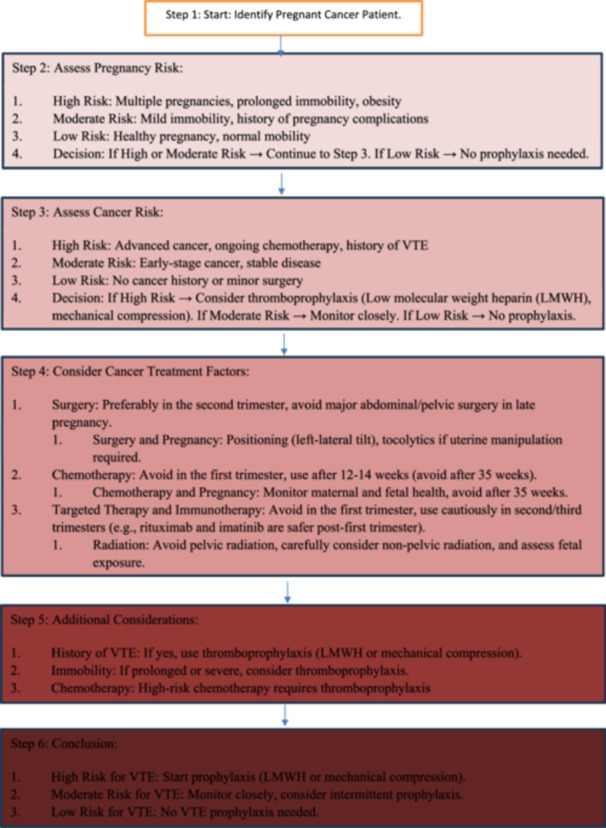
Decision‐making flowchart for VTE prophylaxis in pregnant cancer patients [48].

Figures 2 and 3 summarize the pathophysiology of VTE in the context of pregnancy and cancer as well as decision‐making flowchart for VTE prophylaxis in pregnant cancer patients.

### 4.2 Limitation

This systematic review has several limitations. First, Publication bias is one of the important limitations of this study because studies that report inconclusive or negative results need further investigation. Insufficient demographic data in numerous studies made us unable to comprehensively evaluate the influence of factors such as age, gender, and education on the incidence of VTE. In addition, VTE requires long‐term investigations and one of the limitations was the different and often short follow‐up diversions of the studies. on the other hand, the geographical distribution of the studies included was mainly confined to North America and Europe, potentially leading to an inaccurate representation of global trends in VTE among cancer patients. Furthermore, the diverse approaches to reporting data across the studies made direct comparisons and synthesis of results challenging. Also, a significant proportion of the studies lacked specific details about particular types of cancer and their respective treatments, which are crucial for understanding the complex relationship between cancer characteristics and the risk of VTE.

Another limitation of this study is focusing on English language studies and excluding other languages. Additionally, some studies failed to consider variables that may have some issues to describe VTE risk assessment. The varying methods of diagnosing VTE across the studies create uncertainty regarding the reliability of the reported incidence rates. Furthermore, our review did not investigate the impact of different cancer stages on VTE risk, which shows that more large‐scale studies are needed.

## 5 Conclusion

Evidence in this systematic review showed that VTE and other clotting abnormalities are more likely in pregnant malignancy patients. The treatment of cancer in pregnant women is a major risk feature for VTE. Thromboprophylaxis in hospitalized pregnant females reduces VTE risk.

## Author Contributions

**Ali Vaezi:** writing – original draft. **Seyyed Kiarash Sadat Rafiei:** writing – original draft. **Bita Amiri:** writing – original draft. **Ali Rezvanimehr:** writing – original draft. **Maryam Najiabhary:** writing – original draft. **Pariya Mahdavi:** writing – original draft. **Mohammad Abbasalizadeh:** writing – original draft. **Ghazale Yavari:** writing – original draft. **Mahsa Shirforoush Sattari:** writing – original draft. **Ali Kheirandish:** writing – original draft. **Gisou Erabi:** writing – original draft. **Foad Vakili Zadeh:** writing – original draft. **Fateme Rasekh:** writing – original draft. **Asiyeh Pormehr‐yabandeh:** writing – original draft. **Seyede Zohreh Mohagheghi:** writing – original draft. **Hoda Zaraj:** writing – original draft. **Amir Abdi:** writing – original draft, supervision. **Parisa alsadat Dadkhah:** writing – original draft, supervision. **Niloofar Deravi:** writing – original draft, supervision.

## Ethics Statement

The authors have nothing to report.

## Conflicts of Interest

The authors declare no conflicts of interest.

## Transparency Statement

The lead author Amir Abdi, Parisa alsadat Dadkhah, Niloofar Deravi affirms that this manuscript is an honest, accurate, and transparent account of the study being reported; that no important aspects of the study have been omitted; and that any discrepancies from the study as planned (and, if relevant, registered) have been explained.

## Data Availability

Data is available upon request from corresponding author.
